# Occupational Health Problems among Cambodian Dentists: A Cross-Sectional Study

**DOI:** 10.3390/epidemiologia5030041

**Published:** 2024-09-05

**Authors:** Rodrigo Mariño, Rithvitou Horn, Moniroth Seat, Konitha Hong, Sokpheakta Hen

**Affiliations:** 1 Center for Research in Epidemiology, Economics and Oral Public Health (CIEESPO), Faculty of Dentistry, Universidad de La Frontera, Temuco 4811230, Chile; 2 Melbourne Dental School, The University of Melbourne, Parkville, VIC 3010, Australia; 3 Faculty of Dentistry, University of Puthisastra, Phnom Penh 12211, Cambodia; hrithvitou@puthisastra.edu.kh (R.H.); smoniroth@puthisastra.edu.kh (M.S.); hkonitha@puthisastra.edu.kh (K.H.); hsopheakta@puthisastra.edu.kh (S.H.)

**Keywords:** oral health professions, occupational health, musculoskeletal disorder, Cambodia

## Abstract

Dental practitioners, as part of their work, are exposed to a variety of hazards. This highlights the ongoing need for attention to occupational health in the dental field. A cross-sectional study was organised to investigate the range, prevalence, and associated factors for occupational health problems related to dental practice among Cambodian dentists. Participants underwent a face-to-face interview to explore dentists work-related health problems; 106 Cambodian dentists participated in this study, of which 68.9% were male. Ages ranged from 29 to 71 years, averaging 36.1 years, with the majority (77.4%) in the 29–40 age group. They had 5 to 18 years of practice experience, and worked an average of 52.2 h per week. Commonly reported health issues included back pain (88.7%), headaches (81.1%), shoulder pain (78.3%), arm/hand pain (57.5%), and eye problems (48.1%). Additionally, 38.7% of participants felt stressed and 19.8% depressed. Some reported suicidal thoughts and taking medication for depression. Despite these challenges, 91.5% enjoyed practicing dentistry. These findings highlight the need for interventions and strategies to address the physical and mental well-being of Cambodian dentists. By addressing these issues, steps can be taken to enhance the working conditions and professional satisfaction of dental professionals, ultimately benefiting both the practitioners and their patients.

## 1. Introduction

Occupational health concerns persist across various professions. Despite numerous technological advancements in last few decades [1], dental practitioners continue to encounter multiple occupational health issues. Notwithstanding the implementation of standards and regulations aimed at minimizing health hazards in dentistry, dentists remain susceptible to occupational health problems. These issues encompass a wide range of physical, biological, chemical, radiological, and psychosocial hazards associated with dental practice [2,3,4]. Dental practitioners are also exposed to factors such as excessive noise, vibration, ergonomic stress, as well as occasional instances of workplace and other sources of psychological stress [3,4].

Moreover, dentists often work longer hours in comparison to the standard working week, as highlighted in previous research [1]. This extended workload has been linked to higher rates of depression and even suicide within the dental profession [5,6]. Instances of substance abuse have also been reported among some dentists, including substance dependence. While the majority of dentists report moderate alcohol and/or drug usage [7], it is important note that patterns of alcohol consumption vary significantly among dentists in different countries, mirroring the variations observed in other societal groups [8].

The practice of dentistry, as well as dental training, involves numerous risk factors that necessitate the dental team’s ability to identify and mitigate them to safeguard their own well-being. It is worth noting that individual characteristics can moderate the impact of exposure to these hazards [9]. Nevertheless, research provides evidence that the stresses associated with clinical practice commence during dental education, where the learning environment can be more challenging and hostile compared to other healthcare programs [10,11,12]. 

Fortunately, the negative impacts of stress can be mitigated by a strong personal sense of coherence, exercises, and training, and seeking support from family and friends. Additionally, programs designed to enhance personal confidence can contribute to stress reduction. Furthermore, even dental students can derive benefits from social support networks and vocational orientation programs offered by dental faculties and local professional organisations, as these resources have the potential to alleviate stress [13,14].

Many studies have been conducted regarding the health problems of dentists around the world [15,16,17,18,19,20,21,22,23], however, in Cambodia, no such study has been conducted. Anecdotal reports indicate that Cambodian dentists suffer from a range of health problems, and many dentists have been affected by various illnesses. Some appear to have died prematurely. This study was organised to expand this understanding by investigating the range, prevalence, and distribution, by selected socio-demographic characteristics, work characteristics, and psychosocial factors occupational health problems of Cambodian dentists.

The responsibility for occupational health problems and in acquiring the necessary knowledge to prevent or identify these problems usually falls on dentists themselves and their dental teams. Despite the critical importance of this knowledge, there is often a lack of structured or formal education provided within the profession. Consequently, dentists and their teams must take proactive steps to seek out relevant information, training, and resources to safeguard their health and well-being in the workplace. 

Having prior information on the knowledge, attitude, practice, and beliefs (KAPB) of dentists, strategies at different levels (e.g., dental schools, dental organizations, workplaces, etc.) can be developed to improve awareness, attitudes, and practices would be useful in developing strategies at different levels (e.g., dental schools, dental organizations, workplaces, etc.) to reduce occupational health problems and enhance the well-being of oral healthcare professionals. 

## 2. Materials and Methods

### 2.1. Study Design

In the context of occupational health dentistry, a questionnaire-based cross-sectional survey of Cambodian practicing dentists was organised to assess occupational health among dentists. The conceptual framework adopted in the present study was the knowledge, attitudes, practices, and beliefs (KAPB) approach [24,25]. The KAPB model is widely used in different fields, including occupational health [26,27], to understand and address behaviour change and health promotion. 

Using the KAPB model in the study of occupational health may facilitate understanding and allows insights into the factors that influence occupational health behaviours and can be used to guide the development and implementation of interventions that aim to improve workplace health and safety [25]. 

### 2.2. Sample Size

The appropriate sample size was calculated according to the formula for population-based surveys [28]. Sample calculations indicated that, assuming that 70% of the dental practitioners have experienced occupational health-related problems [17], a sample size of 100 participants yield a confidence level of 0.05 with an estimated effect size between 61% and 79% of dentists have occupational health-related issues.

According to the Country Profile database from the World Health Organization (WHO), in 2018, there were approximately 1395 dentists working in the country [29]. With this number in mind, it was anticipated that these dentists would constitute 7% of the total dental population.

### 2.3. Study Participants (Inclusion and Exclusion Criteria)

Following ethics approval from the University of Puthisastra Research Committee (ID: N31123UP, date of approval: 7 September 2022), a convenience sample of dentists practicing in Phnom Penh, Siem Reap, Kampong Cham, or Kompong Som with at least 5 years of clinical experience, were invited to participate in this study. Traditional dentists, dentists with less than 5 years of experience, and retired dental practitioners were excluded from this study. 

### 2.4. Data Collection Procedures

Three research assistants visited each dentist or spoke to them online. Once the purpose of the study was explained to the dentist, they were invited to provide written consent as for online interviews only after verbal consent was obtained. Consenting dentists underwent a face-to-face or online structured interview with a trained research assistant to explore the work-related health problems that they had experienced during their professional practice, as well as the risk factors associated with them. Data were collected between March 2022 and November 2023. 

### 2.5. Questionnaire Design

The KAPB model was taken into consideration when developing the data collection instrument. The instrument was developed in English based on previous studies [30]. The instrument was then translated into Khmer and back translated from Khmer to English to ensure the meaning of the questions was correct [31]. A pilot study was carried out to ensure that the questions were easy to understand and could be answered with ease. 

The data collection instrument included four sections (See Appendix A):

(1) socio-demographic (i.e., age and sex) and work background information: the location of practice; years of practice; and hours a week practicing dentistry. This section also included a question asking to self-assess their health status as “Poor”, “Fair”, “Good”, or “Excellent”.
(2) Type and severity of eleven occupational health conditions, common to dentistry, that they may be experiencing, including: back pain; headaches; pain in the shoulders, arms, hands, legs, and/or feet; skin problems; eye problems; hearing problems; and neurological problems. These conditions were coded as “Never”, “Sometimes”, and “Often”. Participants were also asked whether they have any transmissible viral infection (e.g., Hepatitis B, Hepatitis C, or HIV). This section also asked about mental health conditions (i.e., stress and depression). A health conditions score was created by adding positive responses (i.e., “Sometimes” and “Often”) across the list of occupational health conditions.
(3) Beliefs about occupational health problems in dental practice. This section included eleven items. The reliability and validity of this occupational health belief scale was reviewed. The construct validity of the scale was assessed through a confirmatory factor analysis of the instrument’s eleven items using the maximum-likelihood estimation method with oblique rotation. The analysis indicated that the factor structure of the instrument had two dimensions: one related to the physical health beliefs, and another related to the mental health beliefs (e.g., stress, depression, level of enjoyment when practicing dentistry). Cronbach’s alpha was used to determine the internal consistency. The reliability of the scale was found to be 0.84. In order to quantify responses, an overall occupational health beliefs index was created by adding the responses across the physical health beliefs items.
(4) Participants were asked whether they believed that occupational health problems in dentistry could be prevented or not. They were also asked whether they had a needle stick injury or not. A positive answer was followed up whether they, as well as the patient, had a blood test.

Additionally, participants were asked whether they have learned the correct position when treating patients and how to reduce stress in dentistry. Participants were presented with a diagram with four positions of the operator doing restorative dentistry, and three positions for the patient.

### 2.6. Statistical Analysis

The statistical analysis provides basic descriptive information on the distribution of the socio-demographic, and work-related variables. To investigate the patterns of responses for Occupational Health scales, Analysis of Variance (ANOVA) were employed. A significant ANOVA was followed by post-hoc comparisons using Tukey’s HSD tests. To test if any combination of the various socio-demographic and work-related variables (e.g., age, sex, location, and year of practice), provided a multivariate explanation of the health conditions scores, a linear regression model was fitted using a stepwise selection method. When a probability value was 0.05 or smaller, the finding was considered to be statistically significant. Data manipulation and analyses were conducted using SPSS PC (Version 25.0, New York, NY, USA).

## 3. Results

A convenience sample of 106 dentists from Phnom Penh (*n* = 41; 38.7%), Kampong Som provinces (*n* = 26; 24.5%), Siem Reap (*n* = 23; 21.7%), and Kampong Cham (*n* = 16; 15.1%) were interviewed. The majority of participants in the study were male (*n* = 73; 68.9%). Ages ranged from 29 to 71 years, with a mean age of 36.1 (s.d. 8.3) years. The majority (77.4%) were in the 29–40 years age group.

Years of practice ranged from 5 to 18 years, with a mean duration of 7.6 (s.d. 3.0) years. The majority (71.7%) had between 5 and 9 years of experience. Participants reported working an average of 52.2 (s.d. 12.9) hours per week, with 31.1% working 56 h a week and another 24.5% working more than 56 h a week. Table 1 shows the socio-demographic work characteristics. Regarding self-reported health status, 60.4% classified their health as “Good” or “Excellent”.

The majority of the participants indicated that they “Often” or “Sometimes” experienced: back problems (88.7%); headaches (81.1%); and pain in their shoulder (78.3%). Over half have pain in their arms and/or hands (57.5%), while almost half of the participants reported (48.1%) eye problems. About one-third of the participants reported either skin problems (30.2%) or leg or foot pain (34.0%). Another 18.8% reported “Often” or “Sometimes” having hearing problems (See Table 2). 

Additionally, 4.7% answered testing positive for blood-borne viruses (i.e., Hepatitis B, Hepatitis C, or HIV). Half of the participants (*n* = 53) have had a needle stick or sharp instrument injury. Of these, about half (*n* = 28: 52.8%) reported that their patients and themselves underwent blood tests. 

Regarding mental health, slightly more than one-third of the participants (38.7%) agreed that they often feel stress when practicing dentistry, and approximately one-fifth (19.8%) said that they often feel depressed and have consulted a doctor or counsellor about the depression they have from practicing dentistry (21.7%). Two dentists reported that they considered committing suicide in the past from work stress and another two reported that they were taken medicines to cope with depression. On the other hand, most participants agreed that they enjoyed practicing dentistry (91.5%). 

In terms of prevention and management of mental health issues, about half of the participants (54.7%) said that they had been taught about how to manage or reduce their stress. Similarly, about half of dentists (50.9%) believed that health problems in dentists could be prevented. 

Overall, participants nominated as an average 6.0 (s.d. 2.3; range 1–11) health problems. There were no significant associations by age, sex, or location. However, there was a statistically significant difference by hours of work (*p* = 0.015), which showed a non-linear association with the number of health conditions. Those in the middle range of 50–70 h a week reported fewer health conditions (5.5; s.d. 2.4) than those in the low (6.3; s.d. 2.1) and higher (8.0; s.d. 1.7) ends. More specifically, regarding musculoskeletal disorders (i.e., back pain, shoulders, legs/feet, or arms/hands), participants nominated as an average 2.6 (s.d. 1.1; range 0–4) problems. However, the number of musculoskeletal conditions was not related to age, hours of work, sex, or time of practice.

In terms of beliefs, the majority of the participants “Agreed” or “Strongly agreed” that practicing dentistry can cause musculoskeletal problems (89.6%) and eye problems (82.1%). Almost half of the participants agreed that practicing dentistry can cause mental health problems (47.2%). Approximately one-quarter of the dentists agreed that practicing dentistry could cause hearing problems, neurological problems, and hypertension. Additionally, more than half of participants (52.8%) agreed that practicing dentistry could cause other (not listed) occupational health problems. On the other hand, about half of participants (49.1%) believed that the dentists’ occupational health problems could be prevented (See Table 3).

When asked about the dentist and the patient positions during treatment, the majority (89.6%) reported being taught the correct positioning of dentist and patient during treatment. However, 66.0% of participants responded that when treating patients, they sit with their thigh parallel to the floor. When asked about dentist-to-patient positions, 71.7% preferred working in a “10–11 o’clock” position followed by 17.0% sitting at a “12 o’clock” position. With regards to patient positioning, 66.0% of the dentists put their patients in a semi-supine position while 29.2% put them in the fully supine position. Five dentists treated their patients sitting up right. 

To test whether any of the SES, KAPB, and work experience variables produced an explanation on the number of self-reported occupational health conditions, particularly musculoskeletal disorders, linear regression analyses were conducted. However, none of the IVs showed evidence of a significant association with the dependent variables (*p* > 0.05) in this study. 

## 4. Discussion

The study identified musculoskeletal disorders, in particular, back problems, shoulder pain, and arm/hand pain, as the most common occupational health condition reported by dentists followed by stress. Participants also reported experiencing various additional occupational health problems, including headaches and eye problems. Risks such as noise and chemical contamination were not reported. Many dentists reported feeling stress or depression from practicing dentistry, and a few even considered suicide. However, most dentists still reported enjoying their practice. 

The relatively high prevalence of stress and depression among participants underscores the importance of addressing mental health concerns in the Cambodian dental profession. It is concerning, however, the number of dentists who reported using medication for their mental health issues. This further emphasises the need to prioritise work-life balance and promote well-being among dentists [20,32]. About half of the participants reported being taught how to manage or reduce stress. Still, most of these findings serve as a call to action for the dental community to address mental health challenges and implement support systems for dentists in Cambodia.

Overall, these findings align with previous research conducted elsewhere, which also reported a high prevalence of musculoskeletal disorders among dentists [15,16,17,18,19,20,21,22,23]. In the same manner, studies report experiences of high levels of work-related stress, and work-life imbalances [20,33]. Reported factors contributing to musculoskeletal disorders include workload, faulty ergonomics, incorrect techniques during patient treatment, and long working hours [20,21,22,23]. In this study, many participants attributed musculoskeletal disorders to improper sitting positions and long work hours. However, in the present results, reported risk factors (e.g., age or sex) were not associated with the number of musculoskeletal disorders or any health conditions experienced. Nevertheless, dentists who worked 50–60 h per week reported fewer health conditions compared to those who worked fewer or more hours. 

Additionally, the study explored the beliefs of dentists regarding the impact of dentistry on their health. It was found that most participants acknowledged the potential for dentistry to cause back problems and eye problems. Furthermore, a significant proportion of dentists agreed that practicing dentistry can lead to mental health problems. This highlights the recognition of the occupational hazards within the profession and the need for preventive measures and support systems.

Effective preventive measures included massage treatments, physical activities, ergonomic equipment, proper working positions, and efficient workflow organization [15,34]. The majority of participants reported sitting in the recommended position. Still, a few dentists provided treatment while standing, which is not recommended according to World Dental Federation ergonomic guidelines [15]. Another third reported positioning patients in positions (e.g., semi-supine), which are not considered optimal according to the World Dental Federation [15]. Working in the correct position can help to avoid musculoskeletal disorders and should be learned and emphasised during the training of dental students.

Additionally, dentists should be aware of the risks of Hepatitis B, Hepatitis C, and HIV in dental settings, and in taking necessary precautions. In the present study, half of participants reported having experienced needle stick or sharp instrument injuries. However, only a minority of participants reported that they and their patients underwent blood tests.

Although this study provides valuable insights into occupational health issues among Cambodian dentists, it is not without limitations. The most obvious limitation was the cross-sectional nature of this study, which precludes a strong conclusion about causal relationships between occupational factors and health outcomes. Another limitation is the self-report nature of the data, which may introduce response bias. Participants may have underreported or overreported their occupational health conditions or mental health problems due to social desirability bias. Furthermore, the study relied on participants’ beliefs about the impact of dentistry on their health, which may not necessarily reflect objective measurements or clinical diagnoses. Additionally, the homogeneity of occupational exposures may have contributed to the lack of statistical significance in the linear regression model.

Still, while this information is useful, it only provides an initial approximation of the true occupational health conditions profile. Findings reflect those conditions among dentists recruited from four locations in Cambodia, therefore generalisation of the study results might be limited. Future research should consider larger, more representative samples and longitudinal designs to gain deeper insights into the occupational health and safety concerns faced by oral health professionals in Cambodia. Further exploration of occupational health by geographic location, and for dentists with longer work experience is needed. Most of the participants were under 50 years of age. Health problems are generally more prevalent in the older age groups. It also appears that some important factors contributing to the development of occupational health conditions were not included in the present study, as none of the variables in the current study were significantly associated with occupational health problems in Cambodian dentists.

Future research should also aim to further investigate the underlying causes of the reported occupational health problems and explore potential interventions that could improve the occupational health and safety of oral health professionals in Cambodia. Further research and interventions in this area can contribute to the development of policies and guidelines that promote a safe and healthy working environment for dental practitioners.

Despite these limitations, the study findings provide valuable insights into the occupational health-related challenges faced by Cambodian dentists. Data were collected using instruments that showed good construct validity and reliability, and present results do not differ greatly from other reports in the literature on occupational health-related challenges faced by dentists.

These findings highlight the importance of addressing the physical and mental well-being of oral health professionals in Cambodia to ensure their continued productivity and job satisfaction. Findings emphasise the importance of dentists to stay updated on new equipment, materials, and techniques that could impact their occupational health, as well as the need to use ergonomic equipment (i.e., saddle seats, loupes, and microscopes) [35] that can help dentists improve their working position and reduce the risk of musculoskeletal disorders [15].

Cambodian dentists should also be equipped with knowledge and resources for effectively managing and seeking support for their mental health conditions and stress. This self-directed approach about prevention and early identification of occupational health concerns underscores the need for greater emphasis on occupational health education within dental curricula and ongoing professional development programs. Continuing education plays a crucial role in enhancing dentists’ awareness and providing practical strategies to prevent occupational hazards and work-related health issues.

Additionally, dental schools should implement comprehensive monitoring systems to safeguard the well-being of their students [13]. It is imperative that information regarding occupational health problems among dentists be incorporated into the undergraduate dental curriculum of all dental schools. Further research is recommended to assess the content of dental school curricula and identify interventions that can promote health and safety in the dental workplace.

## 5. Conclusions

This study sheds light onto the occupational health and safety concerns experienced by Cambodian dentists. It contributes significantly to the existing knowledge gap in this field within the country, emphasising the substantial prevalence of physical and psychological health issues among Cambodian dentists. These findings underscore the urgent need for interventions and support systems to address these concerns and prioritise the well-being of dentists in Cambodia. The outcomes of this study can serve as a foundation for the development and implementation of health and safety policies and interventions aimed at tackling these issues effectively in the future. By gaining a deeper understanding of the knowledge, attitudes, practices, and beliefs of dentists, it becomes possible to mitigate occupational health problems through the implementation of preventive measures, targeted education and training programs, and the identification of barriers or challenges to safety protocols. Addressing these attitudes through awareness campaigns or interventions can promote a safer work environment. Furthermore, evaluating the actual practices of dentists in relation to occupational health measures can identify areas for improvement and inform the implementation of interventions that enhance compliance with safety guidelines.

## Figures and Tables

**Table 1:** Socio-demographic and work characteristics of participating Cambodian dentists.

Variables		
Mean Age (years)	36.1 (s.d. 8.3)	
	Categories	N % ^1^
Sex	Male	73 (68.9%)
	Female	33 (31.1%)
Age group	29–40	22 (20.7%)
	41–50	78 (73.7%)
	Over 50	6 (5.6%)
Year of practicing dentistry	5 to 10 years	89 (84.0%)
	More than 10 years	17 (16.0%)
Place of practice	Phnom Penh	41 (38.7%)
	Kampong Som	26 (24.5%)
	Siem Reap	23 (21.7%)
	Kampong Cham	16 (15.1%)
Working hours (weekly)	Less than 56	47 (44.3%)
	56–70	53 (50.0%)
	More than 70	6 (5.7%)
How would you describe your current health?	Poor/Fair	42 (39.6%)
	Fair/Good	64 (60.4%)

^1^ *n* = 106.

**Table 2:** Occupational health problems reported by Cambodian dentists.

Statement	Never	Sometimes	Often
	N (%)	N (%)	N (%)
I have pain in my back	12 (11.3%)	74 (69.8%)	20 (18.8%)
I have pain in my shoulder(s)	23 (21.7%)	69 (65.1%)	14 (13.2%)
I have pain in my arms and/or hands	45 (42.4%)	54 (50.9%)	7 (6.6%)
I have pain in legs and/or feet	70 (66.0%)	33 (31.1%)	3 (2.8%)
I have a skin problem	74 (69.8%)	31 (29.2%)	1 (0.9%)
I have an eye problem	55 (51.9%)	45 (42.4%)	6 (5.6%)
I have a hearing problem	86 (81.1%)	19 (17.9%)	1 (0.9%)
I have headaches	20 (18.8%)	75 (70.7%)	11 (10.4%)

**Table 3:** Beliefs about occupational health problems in dental practice by Cambodian dentists.

Statement	Agree	Disagree	Do Not Know
	N (%)	N (%)	N (%)
Practicing dentistry can cause musculoskeletal problems	95 (89.6%)	11 (10.4%)	0 (0.00%)
Practicing dentistry can cause skin problems	42 (38.7%)	50 (47.1%)	14 (13.2%)
Practicing dentistry can cause eye problems	87 (82.1%)	13 (12.2%)	6 (5.6%)
Practicing dentistry can cause hearing problems	25 (23.6%)	67 (63.2%)	14 (13.2%)
Practicing dentistry can cause neurological problems	26 (24.5%)	59 (55.6%)	21 (19.8%)
Practicing dentistry can cause mental health problems	50 (47.1%)	37 (34.9%)	19 (17.9%)
Practicing dentistry can cause high blood pressure	25 (23.6%)	54 (50.9%)	27 (25.5%)
Practicing dentistry can cause diabetes	11 (10.3%)	75 (70.7%)	20 (18.8%)
Practicing dentistry can cause other health problems	56 (52.8%)	21 (19.8%)	29 (27.3%)
I enjoy practicing dentistry	97 (91.5%)	3 (2.8%)	6 (5.6%)
Dentists have more health problems than other occupations	30 (28.3%)	45 (42.4%)	31 (29.2%)
I often feel stressed when practicing dentistry	41 (38.8%)	57 (53.7%)	8 (7.5%)
I often feel depressed	21 (19.8%)	72 (67.9%)	13 (12.3%)
Because of work stress and/or depression I have consulted my doctor and/or had counselling	23 (21.7%)	65 (61.3%)	18 (17.0%)
I take medicines to help me cope with my stress and/or depression	2 (1.9%)	96 (90.6%)	8 (7.5%)
Because of work stress and/or depression I have considered committing suicide in the past	2 (1.9%)	103 (97.2%)	1 (0.9%)

## Data Availability

The data that support the findings of this study are available on request from the corresponding author. The data are not publicly available due to privacy or ethical restrictions.

## Appendix A

### Occupational Health Problems among Cambodian Dentists

This survey is confidential, and we will NOT be recording your name on the survey form.

**Part 1: Background information**

1. How old are you? _______ years old
2. Sex:
  ① Female
  ② Male
3. Place of dental practice:
  ① Phnom Penh
  ② Province: ________________
4. How long have you been practicing dentistry? _______ years
5. How many hours do you practice dentistry per week? _______ hours
6. How would you describe your current state of health?
  ① Poor
  ② Fair
  ③ Good
  ④ Excellent

**Part 2: Beliefs about health problems in dentistry**

**Do You Agree or Disagree with the Following Statements?**	**Strongly Disagree**	**Disagree**	**Do Not Know**	**Agree**	**Strongly Agree**
1. Practicing dentistry can cause musculoskeletal problems	①	②	③	④	⑤
2. Practicing dentistry can cause skin problems	①	②	③	④	⑤
3. Practicing dentistry can cause eye problems	①	②	③	④	⑤
4. Practicing dentistry can cause hearing problems	①	②	③	④	⑤
5. Practicing dentistry can cause neurological problems	①	②	③	④	⑤
6. Practicing dentistry can cause mental health problems	①	②	③	④	⑤
7. Practicing dentistry can cause high blood pressure	①	②	③	④	⑤
8. Practicing dentistry can cause diabetes	①	②	③	④	⑤
9. Practicing dentistry can cause cancer	①	②	③	④	⑤
10. Practicing dentistry can cause other health problems. If yes, please specify: ______________________ _________________________________________	①	②	③	④	⑤
11. I enjoy practicing dentistry	①	②	③	④	⑤
12. Dentists have more health problems than other occupations	①	②	③	④	⑤
13. I often feel stressed when practicing dentistry	①	②	③	④	⑤
14. I often feel depressed	①	②	③	④	⑤
15. Because of work stress and/or depression I have consulted my doctor and/or had counselling	①	②	③	④	⑤
16. I take medicines to help me cope with my stress and/or depression	①	②	③	④	⑤
17. Because of work stress and/or depression I have considered committing suicide in the past	①	②	③	④	⑤
18. I feel stressful	①	②	③	④	⑤

**Part 3: Personal health problems**
**Do you have any of the following health problems, and if so, how often?**

**Statement**	**Never**	**Sometimes**		**Often**	**Severity**			
					**Mild**	**Moderate**		**Severe**
1. I have pain in my back	①	②		③	①	②		③
2. I have pain in my shoulder(s)	①	②		③	①	②		③
3. I have pain in my arms and/or hands	①	②		③	①	②		③
4. I have pain in legs and/or feet	①	②		③	①	②		③
5. I have a skin problem	①	②		③	①	②		③
6. I have an eye problem (if yes, describe)	①	②		③	①	②		③
7. I have a hearing problem	①	②		③	①	②		③
8. I have headaches	①	②		③	①	②		③
9. I have other neurological problems (if Yes, please describe) ________________	①	②		③	①	②		③
10. I have a transmissible viral infection e.g., Hepatitis B, Hepatitis C, or HIV	⓪ No		① Yes		⓪ No		① Yes	
11. I have other health problems (if Yes, please list them)	① Yes ② No List: ________________							

**Part 4: Open questions**

12. What do you think are the main reasons some dentists develop health problems?
  ① ___________________________________________________
  ② ___________________________________________________
  ③ ___________________________________________________
  ④ ___________________________________________________
13. Have you ever learned about how to reduce the chance of occupational health problems?
  ① Yes
  ② No
  If yes, where did you learn this? (Please answer all that apply)
  ① Conference
  ② Dental school
  ③ Dental journal
  ④ Other (Please specify) _________________
14. Have you ever been taught the correct positioning of dentist and patient during treatment?
  ① Yes
  ② No
15. Have you ever been taught four-handed dentistry?
  ① Yes
  ② No
16. Have you ever been taught about how to manage or reduce your stress?
  ① Yes
  ② No
17. Have you ever had needle stick or sharps injuries with a contaminated needle or sharp instrument?
  ① Yes
  ② No
  If yes, did you and the patient have a blood test?
  ① Always
  ② Sometimes
  ③ Never
18. Do you think health problems in dentists can be prevented?
  ① Yes
  ② No
  ③ Do not know
  If yes, how?
  ① ___________________________________________________
  ② ___________________________________________________
  ③ ___________________________________________________
19. In the diagram below, which position do you normally sit in when doing restorative treatment?
  ①    □    ②    □    ③    □

20. In the diagram below, which position do you normally sit in when doing restorative treatment?
  A    □    B    □    C    □    D    □

21. In the diagram below, which position is the patient normally in when doing restorative treatment?
  ①    □    ②    □    ③    □    ④    □

22. Which (if any) health problem that you have personally experienced do you think are related to dental practice?
  ① ___________________________________________________
  ② ___________________________________________________
  ③ ___________________________________________________
  ④ ___________________________________________________

**Thank you very much for your time and effort in completing this questionnaire.**
